# Human gastrin- releasing peptide receptor expression in women with uterine cervix cancer

**DOI:** 10.3389/fonc.2023.1126426

**Published:** 2023-01-25

**Authors:** Charles A. Kunos, Denise Fabian, Dana Napier, Mark S. Stonecypher, Ravyn M. Duncan, Jason Hurt

**Affiliations:** ^1^ Department of Radiation Medicine, University of Kentucky, Lexington, KY, United States; ^2^ Biospecimen Procurement & Translational Pathology, University of Kentucky, Lexington, KY, United States; ^3^ Molecular Pathology Laboratory Network, Inc., Maryville, TN, United States; ^4^ Orano Med LLC, Plano, TX, United States

**Keywords:** uterine cervix cancer, cervical cancer, gastrin-releasing peptide (GRP) receptor, uterine cervix adenocarcinoma, radiopharmaceutical

## Abstract

**Introduction:**

^212^Pb-DOTAM-GRPR1 is a pharmaceutical radioimmunoconjugate consisiting of an α-particle-emitting radionuclide lead-212 (^212^Pb), a metal chelator DOTAM (1,4,7,10-tetrakis(carbamoylmethyl)-1,4,7,10-tetraazacyclododecane), and a gastrin-releasing peptide receptor (GRPR)-targeted antagonist currently being evaluated as therapy in uterine cervix and other cancer types. Previous studies have revealed that a variable proportion of uterine cervix cancer tumors overexpress the radiopharmaceutical target GRPR when assessed by cell proportion and staining intensity immunoreactive scores (IRS). Tumor response to ^212^Pb-DOTAM-GRPR1 strongly associates with GRPR overexpression, and therefore, it seems reasonable to assess uterine cervix cancer GRPR immunoreactivity for greater insight into the feasibility of using ^212^Pb-DOTAM-GRPR1 as a radiopharmaceutical treatment.

**Methods:**

We examined a series of 33 uterine cervix cancer paraffin-embedded tumors in order to establish whether this tumor type overexpresses GRPR at an IRS score of 6 or higher, as ^212^Pb-DOTAM-GRPR1 is currently being evaluated in clinical trials against tumors showing such a level of expression.

**Results:**

The results show that five of five (100%) primary adenocarcinomas and 10 of 16 (63%) primary squamous cell tumors overexpress GRPR at an IRS score of 6 or higher.

**Discussion:**

The frequency of overexpression in this study suggests that ^212^Pb-DOTAM-GRPR1 radiopharmaceutical treatment may be useful in the management of persistent, recurrent, or metastatic uterine cervix cancer patients. A phase I clinical trial involving patients with metastatic uterine cervix cancer is currently underway (NCT05283330).

## Introduction

The Commonwealth of Kentucky ranks first in new cases of uterine cervix cancer among United States (US) states, and its women diagnosed with uterine cervix cancer in the Appalachian region drive this observation (12.6 per 100,000 persons (versus 7.7 per 100,000 persons in the total US), age-adjusted to the 2000 US standard population, refs (1, 2).). While early-staged patients are curable, advanced-staged (II–IVB) patients struggle with persistent, recurrent, or metastatic disease, for which treatment remains unsatisfactory. First-line cisplatin-based radiochemotherapy is ineffective in up to 46% of advanced-stage patients (3, 4). Intensifying first-line radiochemotherapy by adding adjuvant surgical intervention is unlikely to raise the cure rate noticeably because of the likelihood of occult metastatic disease (3, 4). Therefore, a reasonable next step would be to evaluate molecularly targeted maintenance therapies attempting better disease cure through control of occult metastatic disease (5).

The strongest etiological factor for uterine cervix cancer tumorigenesis is human papilloma virus infection (6). However, overexpression of the gastrin-releasing peptide receptor (GRPR) might have an important oncogenic role (7–9). GRPR acts as a G-protein-coupled receptor of the bombesin-type family of receptors (10). Its ligand, gastrin-releasing peptide, occurs as a 27-amino acid peptide regulating the physiology of gastrointestinal hormones, smooth muscle cell contraction, and epithelial cell proliferation (10). Its overexpression in uterine cervix cancer and other cancer cell types encouraged the clinical development of a pharmaceutical radioimmunoconjugate ^212^Pb-DOTAM-GRPR1 for the treatment of patients with metastatic or recurrent breast, prostate, colorectal, uterine cervix, melanoma, or non-small cell lung cancers overexpressing GRPR (NCT05283330).

Previous studies have shown that variable subsets of uterine cervix cancer cases expressed GRPR ranging from 77% to 88% as evaluated by diverse methods (7–9). To standardize GRPR reporting, we validated GRPR immunohistochemistry (IHC) staining on an automated IHC staining platform and reported the results using immunoreactive score (IRS) criteria. Characterization of GRPR expression patterns in specific histological subtypes of uterine cervix cancer may help in defining patient population(s) who benefit from ^212^Pb-DOTAM-GRPR1 as a radiopharmaceutical treatment.

## Materials and methods

### Study material

The University of Kentucky Markey Cancer Center and its satellite community oncology practices provide cancer care for a rural agricultural and urban manufacturing region in central and eastern Kentucky (11). Standard hematoxylin–eosin examination under light microscopy determined cell types. Immunoreactivity was assessed in tumors from patients who had previously consented to the future scientific study of their tumors and had their samples stored at the Biospecimen Procurement and Translational Pathology Shared Resource Facility at the University of Kentucky Markey Cancer Center. Twenty-eight primary or metastatic site tumor samples were acquired prior to first-line therapy, whereas five primary tumor samples of persistent disease were taken before any second-line therapy. Thirty-three paraffin-embedded tumors from women with FIGO-staged IA2-IVB invasive uterine cervix cancer were stained for GRPR and reported using IRS scoring criteria (12).

Our primary hypothesis tested whether adenocarcinomas of the uterine cervix had higher IRS expression of GRPR than squamous cell types. Prior to identifying the samples, we made an *a priori* decision to include any patient with available paraffin-embedded uterine cervix cancer primary tumor of the adenocarcinoma or squamous cell type, whether or not paired lymph node or other site metastatic samples were available. This expands our sample size but permits only exploratory descriptive analyses. Data are available from the authors upon reasonable request, with permission from the University of Kentucky Markey Cancer Center. This translational oncology study was approved by the Institutional Review Board at the University of Kentucky (Lexington, Kentucky, #69443).

### Immunohistochemistry

Uterine cervix cancer tumors were sectioned at 4 µm and mounted onto positively charged slides, which were baked at 58° C overnight. Immunohistochemical GRPR staining was performed using the Ventana Benchmark Ultra automated staining platform (Ventana Medical Systems, Tucson, Arizona, USA) per the manufacturer’s instructions. Slides were deparaffinized on the instrument using EZprep Solution (Roche 950-102) for 30 min at 72°C, followed by washing with Ventana Reaction Buffer (Roche 950-300, Basel, Switzerland), which was used for all subsequent washes. Slides then underwent on-board enzymatic antigen retrieval by incubating slides for 4 min with Protease I (Roche 760-2018) followed by washing and incubation with polyclonal GRPR (Origene, TA316872) at a 1:2,000 dilution (0.5 µg/ml) for 52 min at 36°C. The UltraView DAB detection kit (Roche 760-500) was used to visualize antibody staining. Hematoxylin and bluing reagent (Roche 760-2021, 760-2037) were used for counterstain. Slides were dehydrated stepwise through ethanol, then cleared in two exchanges of xylene, and mounted with glass coverslips and Surgipath mounting media (Leica Biosystems, 3801731, Deer Park, Illinois, USA). A positive control colon tissue was included on every run to verify antibody performance.

### Microscopy and immunoreactivity score

**Figure 1:**
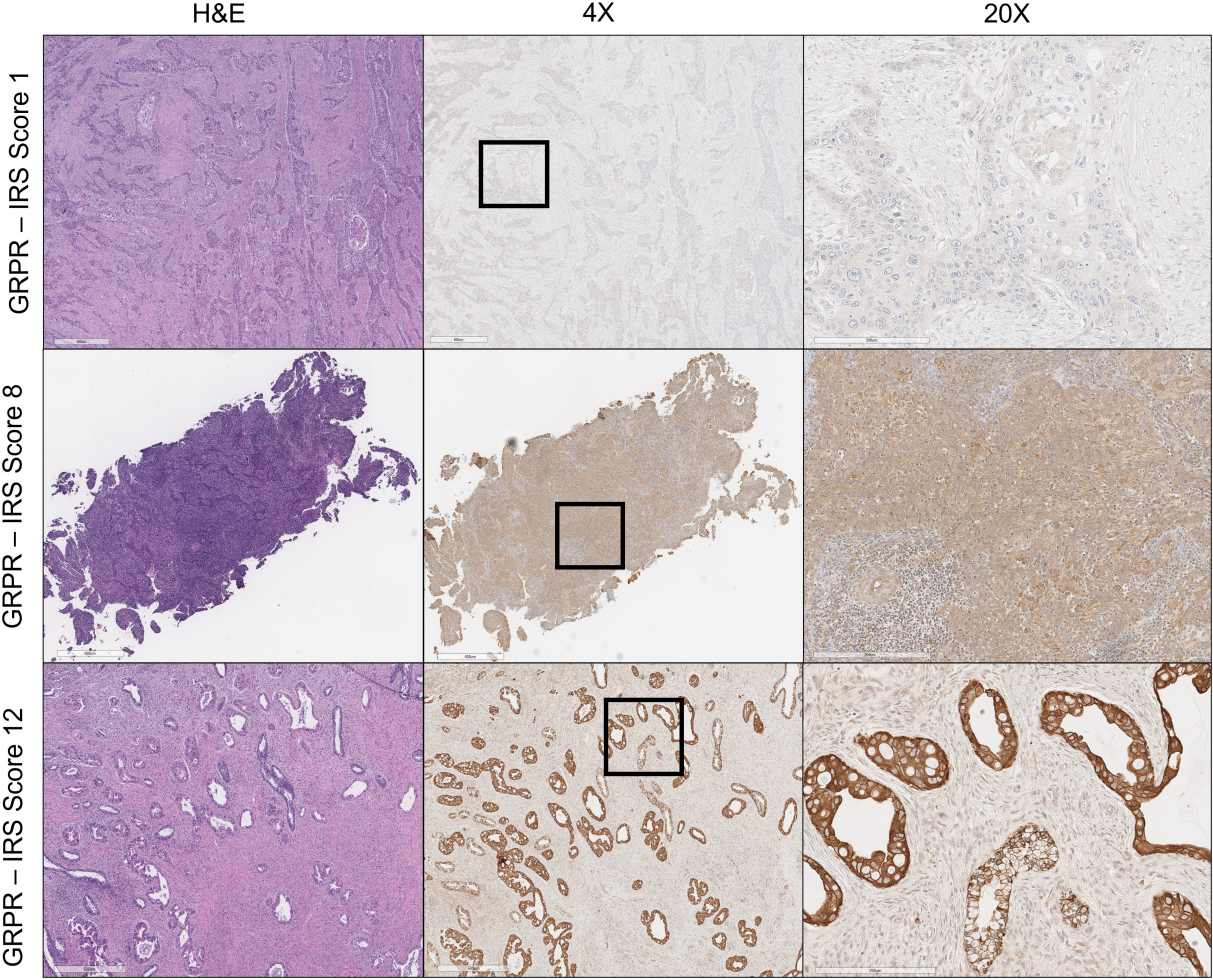
Gastrin- releasing peptide receptor (GRPR) immunoreactivity in uterine cervix cancer. Hematoxylin and eosin (H&E) staining of uterine cervix cancer at ×4 magnification appears in the left column. GRPR staining of uterine cervix cancer at ×4 magnification is in the center, with regions of further magnification boxed. GRPR staining of uterine cervix cancer at ×20 magnification appears in the right column. IRS score is indicated for each row (e.g., percentage of positive cells [4] × intensity of staining [3] = 12: strongly positive).

Individual slides of uterine cervix cancer tumors were viewed on an inverted microscope (Zeiss AxioScope.A1) at ×4–20 magnification (**Figure 1**) . For this pilot project, one histopathologist and one staff scientist blinded to treatment outcome scored the proportion of cells stained and the brown staining intensity of GRPR using a similar reporting format to that described for the interpretation and presentation of immunohistochemistry analysis results (12, 13). Briefly, the IRS has a scoring range of 0–12 due to it being the multiplication product of a subjective percentage positive cell score (0–4) and a subjective staining intensity score (0–3) (**Table 1**). Digital images of uterine cervix cancer microscopy slides were obtained using the Aperio AT2 (Leica Biosystems) scanned at ×40 magnification.

**Table 1:** Immunoreactive score (IRS, adapted from refs (12, 13).

Proportion of positive cells (A)	Intensity of staining (B)	IRS (product of A × B)
0 = no positive cells	0 = no color reaction	0–1 = negative
1 = 0%–10% positive cells	1 = mild reaction	2–3 = mildly positive
2 = 10%–50% positive cells	2 = moderate reaction	4–8 = moderately positive
3 = 51%–80% positive cells	3 = intense reaction	9–12 = strongly positive
4 = 81%–100% positive cells	F**inal IRS (A × B): 0–12**	

## Results

Thirty-three paraffin-embedded tumors were evaluated—21 (64%) primary, six (18%) nodal, and six (18%) metastatic sites. Twenty-eight (85%) tumors were sampled before first-line therapy; five (15%) primary sites of persistent uterine cervix cancer were acquired prior to second-line therapy. Six (18%) patients had paired primary and lymph node metastases available, and thus, 27 individual patients were evaluated (FIGO stage at diagnosis: IA2:1, IB2:1, IIA:1, IIB:8, IIIB:2, IIIC:2, IVA:1, IVB:11). Of the 33 tumors, six (18%) were adenocarcinomas (five primary: one nodal) and 27 (82%) were squamous cell carcinomas (16 primary: five nodal: six metastatic).

### Uterine cervix cancer GRPR immunohistochemistry

GRPR immunoreactivity showed variable degrees of cytoplasmic and cell surface staining among uterine cervix cancer tumors (**Figure 1**). **Table 2** describes the results of GRPR immunostaining for different histopathological features of those tumors. GRPR was immunoreactive at an IRS score of 6 or higher in 20 (61%) of 33 tumors, mostly moderately to strongly diffusely immunoreactive (i.e., 16 (80%) of the 20 tumors scored IRS 8–12). Nine (27%) of 33 tumors were classified as mildly focally immunoreactive (IRS 2–4) and therefore interpreted as mildly positive. Four (12%) of 33 were interpreted as negative (IRS 0–1). GRPR was expressed moderately to strongly immunoreactive in 100% of adenocarcinoma tumors (**Table 2**). Its pattern was moderate to strongly immunoreactive in 52% of squamous cell tumors (**Table 2**). Considering only the primary uterine cervix cancer tumors, all five (100%) adenocarcinomas and 10 (63%) of 16 squamous cell carcinomas had moderately to strongly diffuse immunoreactivity. All five (100%) primary tumors, sampled as persistent disease prior to second-line therapy, scored moderately to strongly diffuse immunoreactive. Four (67%) of six lymph node metastases were mild to strongly positive for GRPR expression. Three (50%) of six other site metastases were moderately positive for GRPR expression.

**Table 2:** GRPR incidence, distribution, and intensity in uterine cervix cancer tumors.

Pathology	GRPR expression				
	Negative	Mild focal	Mild diffuse	Moderate diffuse	Strong diffuse
Adenocarcinoma	0 (0%)	0 (0%)	0 (0%)	3 (50%)	3 (50%)
Primary	0 (0%)	0 (0%)	0 (0%)	3 (60%)	2 (40%)
Node	0 (0%)	0 (0%)	0 (0%)	0 (0%)	1 (100%)
Metastatic	0 (0%)	0 (0%)	0 (0%)	0 (0%)	0 (0%)
Squamous cell	4 (15%)	3 (11%)	6 (22%)	10 (37%)	4 (15%)
Primary	2 (12%)	2 (12%)	2 (12%)	6 (38%)	4 (25%)
Node	1 (20%)	1 (20%)	2 (40%)	1 (20%)	0 (0%)
Metastatic	1 (17%)	0 (0%)	2 (33%)	3 (50%)	0 (0%)

Figures in parentheses are percentages (may not total to 100% due to rounding).

## Discussion

Our team recognizes the importance of molecular triage when selecting a radiopharmaceutical for the treatment of persistent, recurrent, or metastatic uterine cervix cancer (5, 14). Molecular patient enrichment assists in the planning of early-phase clinical trials, as it might influence the overall clinical development of a radiopharmaceutical (15). In this translational oncology study, we tested GRPR protein expression as a potential biomarker to triage uterine cervix cancer patients to a ^212^Pb-DOTAM-GRPR1 radiopharmaceutical treatment.

Molecularly targeted radiopharmaceuticals are being tested in a variety of oncologic settings (14, 15). Because GRPR occurs and stimulates cell proliferation in uterine cervix cancer (7–9), radiopharmaceutical peptide–receptor cohesion might have a therapeutic value by bringing a cell-killing radionuclide directly to cancer cells. The ^212^Pb-DOTAM-GRPR1 radiopharmaceutical is an α-particle-emitting radionuclide ^212^Pb, a metal chelator DOTAM, and a GRPR-targeted antagonist that is currently being studied in the metastatic uterine cervix cancer setting (NCT05283330) as well as in a variety of other cancer types. As the efficacy of this radiopharmaceutical might be predicated upon GRPR overexpression and immunohistochemical enrichment, only patients whose tumors exhibit high (IRS 6–12) score levels are candidates for ^212^Pb-DOTAM-GRPR1 radiopharmaceutical treatment.

Prior reports on patient uterine cervix cancer tumors using nonstandard methods for GRPR expression demonstrated that up to 88% of cancer cases express GRPR and that overall GRPR expression prevails in endocervical neoplasia (7–9). Here, using the trial eligibility-determining IRS assay with corresponding guidelines for evaluation and interpretation, we found concordant results as 71% of primary uterine cervix cancer tumors expressed GRPR. Indeed, by proportion, adenocarcinomas (100%) were more often moderately to strongly immunoreactive than squamous cell cancers (52%). Such a finding fits into the results in other tumor types. For instance, in cutaneous melanoma, 82% of patient tumors were moderately to strongly immunoreactive (16). Elevated levels of GRPR expression are found in the prostate (17), breast (18), colorectal (19), and lung (20) tumors, as assayed by a variety of techniques and scoring criteria.

Currently, it is unknown whether GRPR expression in uterine cervix cancer stays relatively stable during tumorigenesis. For example, 81% of cervical intraepithelial neoplasia (CIN) lesions were moderately to strongly diffusely immunoreactive (7). In the same study, 80% of primary uterine cervix cancers were immunoreactive at the same level (7). Our findings are 71% of primary tumors score moderately to strongly diffusely immunoreactive. Conversely, only half of the metastatic uterine cervix cancer tumors express GRPR at the same level of immunoreactivity; however, such a finding remains exploratory as there were only six metastatic sites sampled and none with a paired primary tumor in our study. Such phenomena need further study and clarification.

In summary, our study suggests that the clinical usefulness of GRPR-targeted radiopharmaceuticals in the maintenance or metastatic treatment of uterine cervix cancer might be beneficial based on the high frequency of GRPR overexpression. It remains desirable to screen uterine cervix cancer patients by GRPR protein expression level as an enrichment approach for clinical trials evaluating the ^212^Pb-DOTAM-GRPR1 radiopharmaceutical.

## Data availability statement

The raw data supporting the conclusions of this article will be made available by the authors, without undue reservation.

## Ethics statement

The studies involving human participants were reviewed and approved by University of Kentucky. The patients/participants provided their written informed consent to participate in this study.

## Author contributions

Authors CK, DF, DN, MS, RD, JH contributed to the design and conduct of the study, interpretation of data, and the writing of this manuscript. All authors contributed to the article and approved the submitted version
